# Severe symptomatic cardiac dysfunction in a patient with *BRAF* V600E-mutated metastatic colorectal cancer treated with encorafenib, binimetinib, and cetuximab: a case report

**DOI:** 10.1186/s40780-025-00480-z

**Published:** 2025-09-01

**Authors:** Masahiro Kondo, Yukiko Nagao, Shohei Hayashi, Eri Wakita, Masato Noda, Itsuki Okada, Chiharu Wachino, Keiko Yamada-Nishide, Masayuki Hori, Yuji Hotta, Yoichi Matsuo, Yoko Furukawa–Hibi

**Affiliations:** 1 Department of Clinical Pharmaceutics, Nagoya City University Graduate School of Medical Sciences, 1-Kawasumi, Mizuho-Cho, Mizuho-Ku, Nagoya, Aichi 467-8601 Japan; 2 Department of Pharmacy, Nagoya City University East Medical Center, 1-2-23 Wakamizu, Chikusa-Ku, Nagoya, Aichi 464-8547 Japan; 3 Department of Gastroenterological Surgery, Nagoya City University Graduate School of Medical Sciences, 1-Kawasumi, Mizuho-Cho, Mizuho-Ku, Nagoya City, Aichi, 467-8601 Japan; 4 Department of Pharmacy, Nagoya City University Hospital, 1-Kawasumi, Mizuho-Cho, Mizuho-Ku, Nagoya, Aichi 467-8602 Japan

**Keywords:** *BRAF* gene mutation, Encorafenib, Binimetinib, Cetuximab, Cardiac dysfunction, Colorectal cancer

## Abstract

**Background:**

V-Raf murine sarcoma viral oncogene homolog B1 (*BRAF*) mutations are present in approximately 5% of Japanese patients with colorectal cancer (CRC) who receive *BRAF*-targeted triplet therapy, consisting of encorafenib (a BRAF inhibitor), binimetinib (a mitogen-activated protein kinase inhibitor [MEKi]), and cetuximab. This combination therapy is associated with an increased risk of cardiac dysfunction (CD), primarily attributed to MEKi. However, the detailed clinical course of this adverse event remains unclear. Here, we report a case of severe symptomatic CD that developed during this triplet therapy.

**Case presentation:**

The patient was a 70-year-old Japanese man diagnosed with *BRAF*-mutated CRC with multiple metastases. *BRAF*-targeted triplet therapy was initiated as a third-line treatment. His baseline left ventricular ejection fraction (LVEF) was 66% and he had no history of heart disease. On Day 106, a pharmacist conducting the patient’s consultation suspected CD associated with binimetinib because of symptoms such as deterioration of general condition and dyspnea. The pharmacist immediately recommended an echocardiography that revealed a significant decline in LVEF to 33%. The patient was referred to a cardiologist and treatment with enalapril, followed by bisoprolol, was initiated while triplet therapy was discontinued. Within 1 week of treatment interruption, the patient’s general condition improved rapidly and his symptoms resolved. Therefore, cancer treatment was resumed as doublet therapy without binimetinib. Under close multidisciplinary monitoring, no recurrence of CD symptoms was observed. Doublet therapy was continued until Day 168, when disease progression occurred. This exceeded the median progression-free survival reported in the phase III BEACON-CRC trial.

**Conclusions:**

This case highlights two crucial insights into BRAF/MEK inhibitor-associated CD. First, even severe symptomatic CD can be effectively managed and reversed upon immediate discontinuation of binimetinib and initiation of cardiotropic medications. Second, in such a severe case, rapid recovery is observed. Once stabilized, *BRAF*-targeted treatment could be continued as doublet therapy without binimetinib to ensure safety and disease control. However, regular echocardiographic surveillance is essential, with an interval shorter than 4 months, based on the clinical course of this case. Additionally, early recognition of CD may be improved by closely monitoring patients’ symptoms and complaints through a multidisciplinary approach.

## Background

Mutations in the v-Raf murine sarcoma viral oncogene homolog B1 (*BRAF*) gene lead to the constitutive activation of the mitogen-activated protein kinase–extracellular signal-regulated kinase (MEK-ERK) signaling pathway, resulting in increased cellular proliferation and growth [1]. *BRAF* mutations, present in 4.5–6.7% of Japanese patients with colorectal cancer (CRC), are associated with poor survival in advanced and recurrent CRC [2, 3]. Most *BRAF* mutations are point mutations at codon 600, where valine (V600) is substituted. These mutations can be effectively targeted with selective *BRAF* inhibitors (BRAFi) [4–8]. Triplet therapy, consisting of encorafenib (ENCO), a BRAFi; binimetinib (BINI), a *MEK* inhibitor (MEKi); and cetuximab (CET), an anti-epidermal growth factor receptor antibody, as well as doublet therapy without BINI, has been approved for the treatment of *BRAF* V600E–mutated metastatic CRC. The phase III BEACON-CRC trial demonstrated that these *BRAF*-targeted combination therapies achieve longer progression-free survival (PFS) and higher overall survival (OS) than standard therapies, including CET and irinotecan [5]. Additionally, this trial reported a trend toward a higher objective response rate in triplet therapy than in doublet therapy.

The combination of BRAFi and MEKi is associated with an increased risk of cardiac dysfunction (CD), including reduced left ventricular ejection fraction (LVEF) or left ventricular dysfunction [4–8]. This pathophysiological mechanism is believed to be primarily associated with MEKi, as cardiovascular toxicity has been reported more frequently in MEKi-containing combinations than in BRAFi monotherapy [4–10]. Phase III trials have documented cardiac adverse events in approximately 4–8% of patients with CRC or melanoma treated with BRAFi and MEKi, with or without CET; however, severe symptomatic cases remain rare [4–8].

Recently, the increasing incidence of cardiovascular diseases (CVD) during and after cancer treatment in patients with cancer is due to several factors, such as the cardiovascular toxicity of cancer therapies [11]. This growing concern has led to the emergence of a new discipline, cardio-oncology, which focuses on the effective management of patients with cancer and CVD [12, 13]. Several clinical guidelines on cardio-oncology have been published, including the 2022 European Society of Cardiology (ESC) guidelines, which are the most recent global guidelines [11, 13–15]. However, information on severe CD associated with BRAFi and MEKi combination therapies remains limited as these are rare adverse events that occur in a small group with *BRAF* gene mutations.

Here, we present the detailed clinical course of a patient with *BRAF* V600E-mutated metastatic CRC who developed severe symptomatic CD during triplet therapy with ENCO, BINI, and CET. Furthermore, based on this case, we discussed the clinical utility of baseline risk assessment and surveillance protocols proposed by the 2022 ESC guidelines.

Patient anonymity and informed consent were obtained in accordance with the Declaration of Helsinki.

## Case presentation

A 70-year-old Japanese man was diagnosed with Stage IVb rectal cancer originating from the appendix, with multiple liver and lung metastases. A *BRAF*-V600E mutation was detected approximately 1 year and a half previously following laparoscopic ileocecal resection. Tests for rat sarcoma virus (*RAS*), mismatch repair (*MMR*) deficiency, and human epidermal growth factor receptor 2 (*HER*2) were negative. The patient received 17 cycles of FOLFOX6 (5-fluoropyrimidine and oxaliplatin) with bevacizumab as first-line treatment, followed by 3 cycles of IRIS [S-1 (tegafur, gimeracil, and oteracil potassium) plus weekly irinotecan] with bevacizumab as second-line treatment. Because disease progression occurred after these regimens, *BRAF*-targeted triplet therapy with ENCO, BINI, and CET was consequently planned as a third-line treatment.

**Fig. 1:**
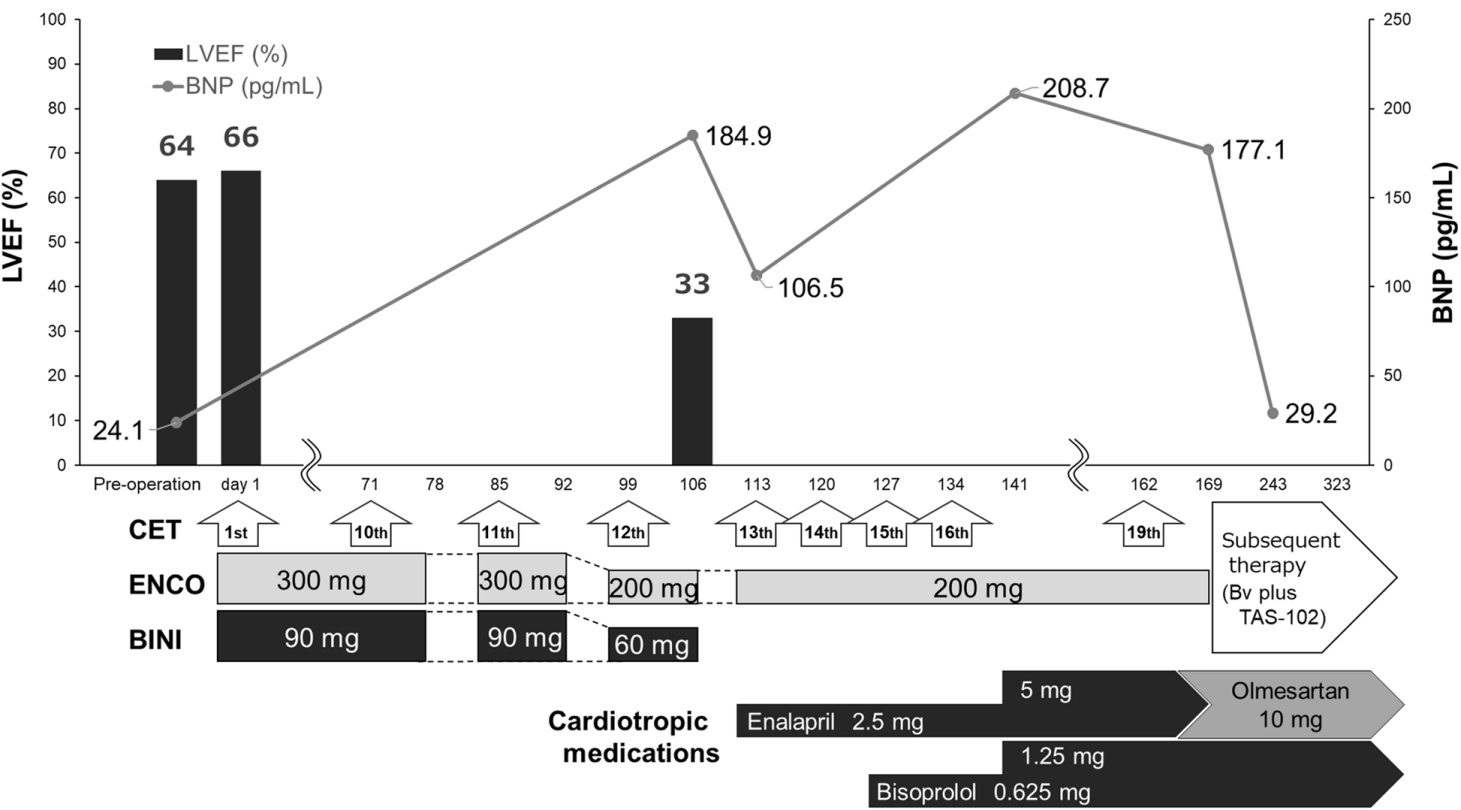
LVEF and BNP during the clinical course of triplet therapy with encorafenib, binimetinib, and cetuximab LVEF, left ventricular ejection fraction; BNP, B-type natriuretic peptide; CET, cetuximab; ENCO, encorafenib; BINI, binimetinib; Bv, bevacizumab; TAS-102, trifluridine/tipiracil hydrochloride

The clinical course of the current case is summarized in Fig. 1 and Table 1. Before initiating triplet therapy, the patient’s Eastern Cooperative Oncology Group performance status (ECOG-PS) was zero. His height was 158.0 cm and his body weight (BW) was 51.4 kg. He had a 30-pack-year smoking history but no history of heart disease. His medical history was notable for hypertension, managed with azilsartan, an angiotensin receptor blocker (ARB). However, he had no trends of polypharmacy and no other comorbidities, including diabetes or dyslipidemia. Additionally, he had no history of prior cardiotoxic cancer therapies. Cardiac assessments demonstrated an LVEF of 64% and a left ventricular internal dimension in diastole (LVDd) of 54 mm preoperatively, which remained stable at 66% and 54 mm, respectively, at baseline. His B-type natriuretic peptide (BNP) level was 24.1 pg/mL and a preoperative 12-lead electrocardiogram (ECG) confirmed a normal sinus rhythm. A chest X-ray performed 7 days before initiating triplet therapy showed no increase in the cardiothoracic ratio (CTR). Baseline liver and renal function tests were within the normal range or classified as grade 1 severity according to the Common Terminology Criteria for Adverse Events (CTCAE) version 5.0. No electrolyte imbalances, including hypokalemia and hypomagnesemia, were observed. Baseline tumor markers were as follows: carcinoembryonic antigen (CEA), 22.5 ng/mL; and carbohydrate antigen 19–9 (CA19-9), 600.1 U/mL.

**Table 1:** Vital signs, body weight, ECOG-PS, renal function data, and associated tumor markers during the clinical course

	**Day**												
	**1**	**50**	**57**	**85**	**99**	**106**	**113**	**120**	**127**	**141**	**148**	**183**	**197**
**Blood pressure** (mmHg)													
** Systolic**	118	110	-	131	124	101	121	122	104	133	-	-	140
** Diastolic**	76	73	-	116	78	73	80	92	60	75	-	-	79
** Heart rate** (beats per minute)	98	114	-	105	73	111	115	122	106	85	-	-	75
** SpO**_**2**_ (%) ^a^	-	97	98	98	98	100	96	97	98	98	-	-	97
** Body temperature** (degree)	36.3	36.4	-	36.3	36.7	-	36.3	36.1	36.2	36.6	-	-	36.2
** Body weight** (kg)	51.4	54.8	-	51.8	51.0	-	49.9	48.5	49.8	50.7	-	-	53.2
** ECOG-PS**	0	0	-	1	2	3	1	1	1	0	-	-	-
**Renal function**													
** Serum creatinine** (mg/dL)	0.76	0.91	0.96	0.83	0.72	1.19	0.76	0.78	0.75	0.78	0.81	0.78	0.86
** eGFR** (mL/min/1.73 m^2^)	77	64	60	70	82	47	77	75	79	75	72	75	67
**Tumor markers**													
** CEA** (ng/mL) ^b^	22.5	-	2.7	3.1	-	-	-	4.0	-	-	12.3	42.6	-
** CA19-9** (U/mL) ^c^	600.1	-	32.8	42.5	-	-	-	56.4	-	-	133.4	619.3	-

*ECOG-PS* Eastern cooperative oncology group-performance status, *eGFR* Estimated glomerular filtration rate, *CEA* Carcinoembryonic antigen, *CA19-9* Carbohydrate antigen 19–9

^a^ Measured on room air

^b^^, c^ The upper limit of the normal range is a) 5.0 ng/mL and b) 37.0 U/mL

On Day 1 of initiating the triplet therapy with ENCO (300 mg daily), BINI (90 mg daily), and CET (400 mg/m^2^, once weekly), no significant abnormalities of vital signs or typical cardiac symptoms were observed. The treatment proceeded as planned, with subsequent weekly CET doses of 250 mg/m^2^. By Day 50, his BW had increased by 3.4 kg from baseline, indicating an increase of 1.8 kg from 53.0 kg recorded on Day −7 (7 days before initiating triplet therapy). However, no deterioration in the patient's general condition or peripheral edema were observed. Furthermore, from Day 22, the patient reported an increase in meal intake. Therefore, the observed increase in BW was suspected to be related to either increased meal intake or variations in the patient's clothing weight, rather than being a sign of CD. Moreover, a remarkable decrease in CEA and CA19-9 levels, the triplet therapy was continued at the same dosage. Two treatment interruptions of all three drugs of the triplet therapy were required from Days 78–85 and Days 92–99 because of the patient’s complaints of anorexia, nausea, or fatigue. However, the treatment was resumed 1 week after each interruption as the symptoms resolved within that period. Additionally, a computed tomography (CT) scan on Day 92 demonstrated no evidence of inflammation, intestinal obstruction, lung abnormalities, or ventricular dilatation. The scan also showed tumor shrinkage, including that of liver metastases. On Day 99, the physician recommended continuing triplet therapy with a dose reduction of ENCO (200 mg daily) and BINI (60 mg daily). However, before the administration of intravenous CET, a pharmacist assessing the patient at the bedside noted a significant decline in general condition (ECOG-PS 2) and the presence of exertional dyspnea with stable SpO_2_ (98% on room air). Based on these clinical findings, the pharmacist suspected cardiovascular adverse events related to ENCO and BINI or BINI alone, rather than pulmonary disease, and immediately recommended echocardiography. However, the echocardiography was scheduled for the following week and the triplet therapy, including ENCO and BINI, was continued based on the physician’s judgment, considering the balance between treatment efficacy and safety. The following week (Day 106), echocardiography revealed a significant decline in LVEF to 33%, classified as grade 3 in severity according to CTCAE, with an LVDd of 44 mm. Additionally, the patient’s general condition deteriorated further (ECOG-PS 3). Despite this, ECG findings exhibited no abnormalities, including QT interval prolongation (QTs 434 ms). No hypokalemia or hypomagnesemia were observed on this day. Additionally, BNP level increased to 184.9 pg/mL, exceeding the upper limit of normal (18.4 pg/mL). During this period, renal function data transiently worsened to grade 1 (CTCAE) but recovered in the following week and subsequently remained within the normal range. Given these results, the physician and pharmacist decided to interrupt all three drugs from Day 106 and refer the patient to a cardiologist. The cardiologist diagnosed the condition as CD primarily caused by BINI and initiated treatment with enalapril (2.5 mg daily), an angiotensin-converting enzyme inhibitor. On Day 113, 1 week after treatment interruption, cancer treatment was resumed as doublet therapy combining ENCO with CET, while BINI was permanently discontinued. This decision was based on the prompt recovery of the general condition (ECOG-PS 1) and resolution of dyspnea. Since then, the cardiologist continued regular follow-ups. On Day 127, the chest X-ray image showed no change or only a slight decrease in the CTR compared to the previous assessment, as evaluated by the cardiologist. Bisoprolol (0.625 mg daily), a beta-blocker, was introduced and the dosage was increased to 1.25 mg daily from Day 141. Additionally, enalapril (2.5 mg daily) was increased to 5.0 mg daily but was later switched to olmesartan (10 mg daily), an ARB, owing to elevated blood pressure. Throughout both triplet therapy and subsequent doublet therapy, hypokalemia was observed once on Day 99, and hypomagnesemia on Days 85, 99, 113, and 120. Nevertheless, these events were transient and of grade 1 severity (CTCAE ver. 5.0). Hypomagnesemia was appropriately corrected via magnesium sulfate supplementation. No recurrence of CD symptoms occurred throughout the treatment duration. However, disease progression, indicated by tumor enlargement in liver metastases, was observed on a CT scan on Day 157. Doublet therapy was continued until Day 168 before transitioning to subsequent treatment.

## Discussion and Conclusions

To the best of our knowledge, this is the first report to fully document the clinical course of severe symptomatic CD during *BRAF*-targeted triplet therapy with ENCO, BINI, and CET. This report provides clinically valuable information on this treatment-related adverse event, including onset timing, reversibility, recovery duration, therapeutic response to cardiotropic medications, and impacts on cancer treatment.

Among them, this report highlights that CD is reversible even in a severe symptomatic case and indicates the specific duration required for recovery after BINI discontinuation. Additionally, this report highlighted the crucial role of pharmacist interventions in the early recognition of adverse events and prevention of severe outcomes through a multidisciplinary approach.

CD is a well-established side effect associated with certain combination therapies of BRAFi and MEKi, including ENCO and BINI with or without CET [4–8, 16–20]. However, severe symptomatic presentations are uncommon. Data from phase III trials and two retrospective real-world studies have reported the incidence of symptomatic or grade 3/4 CD (according to CTCAE) to be approximately 0–4% (Table 2A, B) [4–8, 17, 18]. Moreover, these randomized phase III trials identified a consistent trend in which the incidence of CD was higher in the combination arm with MEKi than in the BRAFi monotherapy arm. Based on these findings, the study protocols recommend interrupting or permanently discontinuing MEKi alone in patients who develop CD during treatment. Following this approach, we discontinued only BINI (a MEKi) while continuing ENCO with CET as doublet therapy. CD did not recur at any point after the switch, strongly indicating that BINI alone was the primary contributor to cardiac dysfunction. Additionally, CT scans to assess disease progression were performed every 2–3 months, an interval considered appropriate in clinical practice. Consequently, the PFS exceeded 5 months, which was longer than the median PFS reported in the phase III trial for patients with metastatic CRC (4.3 months in the triplet therapy arm with ENCO, BINI, and CET and 4.2 months in the doublet therapy arm with ENCO and CET) [5]. Thus, continuing *BRAF*-targeted doublet therapy without BINI after the development of CD appears to be an appropriate approach in terms of safety and disease control. A prior retrospective study on BRAFi and MEKi combination therapy reported no correlation between a decrease in LVEF during treatment and PFS or OS. Therefore, the outcome in the current case is consistent with these findings [18].

**Table 2:** Review of previous reports of cardiac dysfunction associated with BRAF and MEK inhibitor combination therapy

**A.** Phase III clinical trials (randomized studies)						
					**Incidence of cardiac dysfunction**^**c**^**(%)**	
**Ref.** **No.**	**Acronym**	**Cancer type**	**BRAF and MEK inhibitors (daily dosage)**^**a**^	**No. of cases**^**b**^	**Any grade**^**d**^	**Grade 3–4**^**d**^
[4]	COLUMBUS	Melanoma	ENCO (450 mg) + BINI (90 mg)	192	8.0	2.0
			ENCO (300 mg)	192	2.0	1.0
			VEM (1,920 mg)	186	1.0	0.0
[5]	BEACON-CRC	Colorectal Cancer	ENCO (300 mg) + BINI (90 mg) + CET	222	4.0	NA
			ENCO (300 mg) + CET	216	0.0	0.0
[6, 7]	COMBI-d	Melanoma	DAB (300 mg) + TRA (2 mg)	209	4.0	1.0
			DAB (300 mg)	211	3.0	2.0
[8]	COMBI-v	Melanoma	DAB (300 mg) + TRA (2 mg)	350	8.0	4.0
			VEM (1,920 mg)	349	0.0	0.0
B. Retrospective studies						
				**Cardiac dysfunction**		
**Ref.** **No.**	**Cancer type**	**BRAF and MEK inhibitors** **(daily dosage)**	**No. of** **cases**	**definitions**	**Incidence (%)**	**Onset timing**^n^
[16]	Melanoma	ENCO (450 mg) + BINI (90 mg)^e^	108	Minor cardiotoxicity^h^	18	Median: 78 days (range: 71–1246)
				Major cardiotoxicity^i^	6	Median: 134 days (range: 76–377)
[17]	Melanoma	DAB (NA) + TRA (NA) or DAB (NA) + TRA (NA) followed by ENCO (NA) + BINI (NA) or DAB (NA) followed by ENCO (NA) + BINI (NA)^f^	63	Mild ^j^	17.5	73% occurred at 4 weeks 18% occurred at 4 months
				Moderate ^k^	9.5	50% occurred at 4 weeks 33% occurred at 4 months
				Severe (Grade >=3) ^d, l^	0.0	NA
[18]	Melanoma	VEM (NA) + COBI (NA) or DAB (NA) + TRA (NA) or BRAFi or MEKi monotherapy^g^	88	decrease in LVEF^m^	13.6 (Grade 2: 11.4, Grade 3: 2.3)^d^	Median: 11 months (IQR: 3–21)
**C.** Case reports						
				**Cardiac dysfunction**		
**Ref.** **No.**	**Cancer type**	**BRAF and MEK inhibitors** **(daily dosage)**	**Age** **/Sex**	**Event names**	**Severity**^d^	**Onset timing** ^j^
[19]	Melanoma	DAB (NA) + TRA (NA)	52 y /Male	pericardial effusion cardiac tamponade	Grade 4	14 days
[20]	Melanoma	DAB (NA) + TRA (NA)	69 y /Male	decrease in LVEF	Grade 3	30 days
Current case	Colorectal cancer	ENCO (300 mg) + BINI (90 mg) + CET	70 y /Male	decrease in LVEF	Grade 3	106 days

*ENCO* Encorafenib, *BINI* Binimetinib, *VEM* Vemurafenib, *CET* Cetuximab, *DAB* Dabrafenib, *TRA* Trametinib, *NA* Not applicable, *BRAFi* BRAF inhibitor, *MEKi* MEK inhibitor, *COBI* Cobimetinib, *IQR* Interquartile range, *LVEF* Left ventricular ejection fraction, *GLS* Global longitudinal strain

^a^Control arms other than BRAFi or MEKi are omitted

^b^Safety analytic cases in each study

^c^Adverse cardiovascular events associated with left-ventricular dysfunction (Ref. Nos. 5 and 6) or a decrease in the ejection fraction (ref. No. 7–9)

^d^Common Terminology Criteria for Adverse Events v4.0 or 5.0

^e^Includes three patients who were treated with dose reduction of both drugs to 66% due to baseline LVEF<50%.

^f^Patients received DAB +TRA (*n*=54); DAB +TRA followed by ENCO + BINI (*n*=8); or DAB followed by ENCO + BINI (*n*=1)

^g^Patients received VEM +COBI (*n*=40); DAB+TRA (*n*=29); BRAFi monotherapy (VEM, DAB, ENCO; n=18); or MEKi monotherapy (BINI; *n*=1)

^h^Defined as LVEF reduction of ≥15 percentage points but remaining >50%

^i^Defined as a ≥10 percentage point decline in LVEF to <50%

^j^Defined as GLS worsening >15% relative to baseline with LVEF remaining ≥50%

^k^Defined as a reduction in LVEF to 40%–49% + either ≥10% LVEF reduction from baseline or GLS worsening by >15% relative to baseline

^l^Defined as LVEF reduction to <40%

^m^Defined as a ≥10 percentage point decline in LVEF to <55%

^n^Duration from initiation of BRAF and MEK inhibitors

In this patient, ECOG-PS significantly improved after the interruption of BINI because of a decrease in LVEF and the long-term treatment interruption was not necessary. Additionally, echocardiography was performed based on a pharmacist’s recommendation, leading to timely diagnosis and intervention by a cardiologist. These findings suggest that this treatment-related CD, even in severe cases, can be effectively managed through early detection and immediate intervention. A strong multidisciplinary approach can play a crucial role in achieving these outcomes. Such coordinated efforts may help minimize treatment interruption and ultimately prolong PFS. In the present case, the decision to resume doublet therapy was based on the recovery of ECOG-PS and resolution of the patient’s symptoms, without performing echocardiography. No recurrence of CD was observed following resumption. This suggests that ECOG-PS and patient symptoms serve as practical indicators for restarting BRAF-targeted doublet therapy after the development of CD, provided there is close monitoring and intervention from a multifaceted perspective by a multidisciplinary team, including physicians, cardiologists, and pharmacists.

Regarding the timing of severe CD onset after initiating the combination therapy of BRAFi and MEKi with or without CET, two retrospective studies reported median onset times of 134 days (range: 76–377 days) and 11 months (interquartile range: 3–21) [16, 18]. Another study reported that 50% of patients developed moderate CD within approximately 4 weeks [17] (Table 2B). Additionally, two case reports described CD onset at 14 days and 30 days [19, 20] (Table 2C). In this case, a significant decrease in LVEF was observed on Day 106. Based on these findings, CD onset in clinical practice should typically be expected within 4 months, considering the severity and potential for serious outcomes. However, accurately determining the onset remains challenging owing to limited data and considerable variability among cases. Differences in the frequency of echocardiography, CD definitions, LVEF measurement modalities, and BRAFi/MEKi regimens may have affected the reported onset times in previous studies. Therefore, although these findings provide useful insights, they should be interpreted with caution and further investigation is warranted. Considering these factors is expected to contribute to establishing appropriate schedules for echocardiographic surveillance of CD in the future.

Baseline cardiovascular risk may affect the incidence and timing of CD onset. A systematic review and meta-analysis, which included 2,317 patients from five randomized controlled trials on melanoma, investigated risk factors for CD associated with BRAFi and MEKi combination therapy [21]. This study revealed that patients younger than 55 years have a higher risk of decreased LVEF. However, our current case did not meet this criterion. Additionally, differences in treatment regimens, such as the inclusion of CET or variations in cancer types, do not appear to significantly impact cardiovascular risk, although CET is known to increase the risk of hypomagnesemia [5]. No notable differences in CD incidence have been identified across multiple phase III trials in patients with melanoma or CRC treated with various BRAFi/MEKi regimens, with or without CET. In contrast, the 2022 ESC guidelines on cardio-oncology recommend assessing pretreatment CVD risk using the Heart Failure Association–International Cardio-Oncology Society risk assessment tools. These guidelines also suggest implementing cardiovascular surveillance during BRAFi and MEKi therapy based on each patient’s stratified risk level [13, 22]. Table 3 presents the results of a retrospective application of baseline cardiovascular toxicity risk stratification to the current patient, following the recommendations of this guideline. The patient was estimated to have accumulated more than five points of moderate-risk factors (Table 3A), classifying him as “high-risk” (Table 3B). This suggests that risk assessment and stratification based on these guidelines are valuable for predicting CD, despite a prior report indicating limitations in the utility of this risk stratification tool [17]. For high-risk patients receiving BRAFi and MEKi combination therapy, the guidelines recommend considering echocardiographic surveillance every 4 months during the first year. However, in this case, CD developed within 4 months, as previously discussed, and several prior reports have also documented onset within this timeframe [17, 19, 20]. Therefore, for high-risk patients, echocardiographic surveillance for CD may be performed at intervals shorter than 4 months to ensure early detection and timely intervention.

**Table 3:** Baseline cardiovascular toxicity risk stratification in patients receiving BRAF and MEK inhibitor combination therapy

A. Baseline risk assessment		
**Baseline cardiovascular toxicity risk factors**	**Risk level**	**Current case**
**Previous CVD**		
HF/cardiomyopathy/CTRCD	VH	
Severe VHD	H	
MI or PCI or CABG	H	
Stable angina	H	
Arrhythmia^a^	M1	
**Cardiac imaging**		
LVEF < 50%	H	
LVEF 50–54%	M2	
**Cardiac biomarkers**^b^		
Elevated baseline cTn	M2	
Elevated baseline NP	M2	**✓**
**Age and CVRF**		
Age ≥ 80 years	M1	
Age 65–79 years	M1	**✓**
Hypertension^c^	M2	**✓**
Chronic kidney disease^d^	M1	
DM	M1	
**Previous exposure to**		
Anthracycline	H	
RT to left chest or mediastinum	M2	
**Lifestyle risk factors**		
Current smoker or significant smoking history	M1	**✓**
Obesity (BMI > 30 kg/m^2^)	M1	
**B.** Risk stratification		
**Risk stratification**	**Definitions**	**Current case**
**Low risk**	no risk factors OR one M1 risk factor	
**Moderate risk**	moderate risk factors with a total of 2–4 points (M1 = 1 point; M2 = 2 points)	
**High risk**	moderate risk factors with a total of ≥ 5 points OR any high-risk factor	**✓**
**Very high risk**	any very high risk factor	

*CVD* Cardiovascular disease, *HFA-ICOS* Heart failure association international cardio-oncology society, *HF* Heart failure, *CTRCD* Cancer therapy-related cardiac dysfunction, *VHD* Valvular heart disease, *MI *Myocardial infarction, *PCI* Percutaneous coronary intervention, *CABG* Coronary artery bypass graft, *LVEF* Left ventricular ejection fraction, *cTn* Cardiac troponin, *NP* Natriuretic peptides, *DM* Diabetes mellitus, *RT* Radiotherapy, *BMI* Body mass index, *VH* Very-high risk, *H* High risk, *M1* Moderate 1, *M2* Moderate 2

^a^Atrial flutter, ventricular tachycardia, or ventricular fibrillation

^b^Elevated above the upper limit of the normal local laboratory reference range

^c^Systolic BP > 140 mmHg, diastolic BP > 90 mmHg, or on treatment

^d^eGFR < 60 mL/min/1.73 m^2^; f, HbA1c > 7.0% or > 53 mmol/mol, or on treatment

For this patient, we elected to perform echocardiographic surveillance for CD 4 months after initiating triplet therapy, based on the aforementioned recommendation of the 2022 ESC guidelines [13]. However, this planned schedule may be insufficient as a severe symptomatic CD developed just prior to the echocardiography. This indicates a need for more proactive surveillance. In contrast, some clinical trials in melanoma patients have adopted an echocardiographic surveillance schedule involving repetitions at 4 weeks and every 12 weeks thereafter [6–8]. This approach has also been supported by a prior report [23]. Furthermore, the Phase III BEACON-CRC trial, which is directly relevant to our current case, also adopted this same schedule [5]. While several proposals or recommendations for CD surveillance procedures currently exist, a definitive consensus has yet to be established. In this context, our case highlights the potential benefits of implementing echocardiography early on for detecting CD promptly, especially given that CD is designated as a “critical identified risk” in the Risk Management Plan of BINI in Japan. Moreover, an individualized approach that considers each patient's clinical course, including weight gain during treatment, may also be necessary. Consequently, further investigation is warranted to determine the appropriate schedule of echocardiographic surveillance for CD, informed by these various proposals and our current report.

This report is the first to provide a detailed account of the clinical course of severe symptomatic CD associated with *BRAF*-targeted triplet therapy using ENCO, BINI, and CET for metastatic CRC. This treatment-related CD was promptly reversed following the immediate discontinuation of BINI (a MEKi) and initiation of cardiotropic medications, even in severe cases. Therefore, continuing *BRAF*-targeted doublet therapy without BINI may be a viable approach for maintaining safety and disease control. However, predicting the onset of CD remains challenging owing to limited data. Based on the available evidence, CD should be assumed to occur within 4 months in clinical practice, necessitating regular echocardiographic surveillance. Additionally, early recognition of CD symptoms may be improved by carefully assessing patient-reported complaints from a multidisciplinary perspective. We highlighted the crucial role of pharmacist interventions in this regard. While these may be considered general clinical management strategies, there are no prior reports demonstrating the utility of such interventions for CD from this perspective. Relying solely on echocardiographic monitoring may be insufficient owing to limitations in frequency and interval. Thus, discussions concerning close monitoring through a multidisciplinary approach are crucial.

## Data Availability

No datasets were generated or analysed during the current study.

## Abbreviations

ARB: Angiotensin receptor blocker
BINI: Binimetinib
BRAFi: BRAF inhibitor
BW: Body weight
CA19-9: Carbohydrate antigen 19–9
CD: Cardiac dysfunction
CEA: Carcinoembryonic antigen
CET: Cetuximab
CRC: Colorectal cancer
CRP: C-reactive protein
CT: Computed tomography
CVD: Cardiovascular diseases
ECG: Electrocardiogram
ECOG-PS: Eastern Cooperative Oncology Group performance status
ENCO: Encorafenib
LVDd: Left ventricular internal dimension in diastole
LVEF: Left ventricular ejection fraction
MEKi: MEK inhibitor
OS: Overall survival
PFS: Progression-free survival
